# Research and Remedies

**Published:** 1912-08-31

**Authors:** 


### RESEARCH AND REMEDIES.

### Acute Appendicitis.

Finality seems as far away as ever in the vexed question of when to operate in acute appendicitis, notwithstanding the fact that three surgeons out of four seem to have laid down at some time or other hard-and-fast principles for the guidance of their colleagues. At the Liverpool meeting of the British Medical Association this evergreen topic was introduced by Mr. H. J. Paterson, who is one of those favouring immediate operation in all cases without exception. He produced a series of cases treated on these lines with a mortality of just over 3 per cent., and challenged those who taught differently to produce figures as good. This challenge was taken up by Sir G. Beatson, whose figures certainly showed a record no jot inferior to Mr. Paterson's. In the discussion most of those present sided with the latter, but Sir George had some support for his views upon the treatment of late cases with localised mischief. Considering that this question has been dividing surgical opinion now for fully a decade, it is remarkable that it is not yet settled; probably the truth is that each system has drawbacks which lead to occasional failures.

### The Control of the Suprarenal Glands.

The ductless glands have received much attention of late years, but they still afford countless problems as yet quite unsolved. In the *Journal of Physiology* Dr. T. R. Elliott, who is a clinician as well as a physiologist, contributes a paper on the subject of the control of the suprarenal glands by the splanchnic nerves. The amount of adrenalin produced is the same on both sides. Morphia and ordinary anæsthesia with ether, chloroform, and urethane cause exhaustion of adrenalin. Excitation of afferent nerves (such as great sciatic), or direct injury to the brain, also causes loss of adrenalin; the centre controlling this loss is close to the vasomotor centres in the bulb; and the efferent path is by the splanchnic sympathetic nerves. If these latter are cut, exhaustion does not occur; and in like circumstances ether, chloroform, pilocarpine, and diphtheria toxin have no effect, from which it is concluded that they have no direct effect upon the suprarenals. Electrical stimulation of the splanchnic nerves discharges adrenalin into the blood, with fall of blood pressure. These researches may be found to bear upon some of the problems of anæsthesia.

